# Use of knowledge translation products from health technology assessment: a prospective observational study

**DOI:** 10.1017/S0266462325103371

**Published:** 2026-01-09

**Authors:** Ashkan Baradaran, Nicolas Parenteau, Isabelle Ganache, Olivier Demers-Payette, Mélanie Martin, Yannick Auclair, Roland Grad, Pierre Pluye

**Affiliations:** 1 Department of Family Medicine, McGill University Faculty of Medicine, Canada; 2 Institut national d’excellence en santé et en services sociaux, Montréal, Canada

**Keywords:** information assessment method, health technology assessment, knowledge translation, survey, knowledge products

## Abstract

**Background:**

Few studies report the evaluation of the use of Health Technology Assessment (HTA) organizations’ knowledge products.

**Objectives:**

To determine (a) the stakeholders’ use of the products disseminated by the ‘*Institut national d’excellence en santé et en services sociaux’* (INESSS), and (b) the variability of use according to user characteristics and product properties.

**Methods:**

A prospective web survey was performed. We included all participants who accessed INESSS products and voluntarily completed an online questionnaire from 1 January 2021, to 31 December 2022. For each rated product, the participants’ use and intention to use were documented using the content-validated Information Assessment Method (IAM) questionnaire. Descriptive statistical analyses were conducted.

**Results:**

A total of 7041 responses were gathered. After removing incomplete and ineligible responses, we were left with 5236 responses; 74.4 percent of responses were from women; 5014 (95.8 percent) reported that the product was relevant; of those, 4322 (82.5 percent) indicated that the respondent was satisfied; of those, 4096 (78.2 percent) reported that the product was used or had an intention to use the product. Regarding products’ use (*n* = 3023; 57.7 percent), there was no difference between regions with versus without medical faculties. Older participants were less likely to report using a product. Products with recommendations were more likely to be used, and healthcare professionals were more likely to use the products compared to other participants.

**Conclusions:**

Current findings help identify audiences for targeted dissemination, guide user engagement strategies, and inform product refinement. Recommendation-containing products show the greatest uptake, particularly among younger professionals.

## Introduction

The literature on the evaluation of Health Technology Assessment (HTA) organizations suggests that the continuous quality assessment of HTA knowledge products can improve their outcomes and the production process (1). The Information Assessment Method (IAM) is a content-validated method to evaluate knowledge products (2). IAM users have included health managers, nurses, patients, parents of young children, pharmacists, physicians, residents, rehabilitation professionals, and other health information consumers. The IAM is implemented in more than twenty-five projects in Belgium, Brazil, Canada, and the United States. The IAM questionnaire allows users of knowledge products to document outcomes (relevance, cognitive impact, intention to use, and expected benefits) of information delivered by or gained from digital sources. Research has demonstrated IAM’s utility in documenting the influence of information on decision-making and its subsequent health outcomes (3;4). For example, the ecological content validation of IAM for parents highlighted its value in assessing information from a user’s perspective, ensuring relevance and actionable insights (5). Additionally, the method has been validated through mixed-methods research to enhance its content and applicability (6). Over the past 15 years, IAM has evolved to include applications such as electronic knowledge resources and patient education materials, providing a systematic approach to evaluating health-related information (2). These studies collectively underscore the versatility and significance of IAM in advancing the evaluation of knowledge products.

INESSS was created in 2011, merging the *Conseil du médicament and the Agence d’évaluation des technologies et des modes d’intervention en santé* (AETMIS). The mission of INESSS is to promote clinical excellence and the efficient use of resources in the health and social services sector. INESSS aims to help and support decision-making and improve practices (www.inesss.qc.ca/en/about-us/about-the-institut.html). In 2018, the *Institut national d’excellence en santé et en services sociaux* (INESSS) implemented the IAM questionnaire to assess its knowledge products.

The purpose of this study is to analyze the use of INESSS products. Considering that INESSS reports present descriptive statistics (7;8), it was decided that this protocol should focus on inferential statistics. Thus, our specific quantitative research questions are as follows: (a) To what extent do IAM respondents use the products disseminated by INESSS? (b) To what extent does the product use vary according to user characteristics and product properties?

## Methods

### Study design

We conducted a prospective observational study and reported the methods in accordance with the Consensus-Based Checklist for Reporting of Survey Studies (CROSS checklist) (9).

### Instrument

We used the IAM questionnaire (4;5). In the first part of the IAM questionnaire, for demographic data, each participant was asked closed-ended questions about the type of product, application, gender, age group, occupation, and region. The second part of the questionnaire consisted of closed-ended questions that document users’ satisfaction regarding a product, its relevance, use, and intention to use (see IAM-INESSS-2019 version in Supplementary Appendix 1). The closed-ended questions were answered in a sequential manner. If they did not find the product relevant or were not satisfied with the product, they were not asked the rest of the questions. If they had used the product before, they were not asked about their intention to use it in the future. The last part of the questionnaire was dedicated to open-ended questions that asked participants to reflect on what they appreciated more about the product, how it could be improved, and what other suggestions they might have.

### Data collection

INESSS started gathering data, pilot testing, and perfecting the implementation of the data gathering from 2018. In this study, responses from 1 January 2021 to 31 December 2022, will be analyzed (“pull” data). Participants looked for information on the INESSS web site, accessed INESSS’s project webpage, opened an INESSS product, and completed an online IAM questionnaire (2). The list of variables is presented in Supplementary Appendix 2. The questionnaire was available online when stakeholders visited the knowledge products webpage. The questionnaire was accessible to all stakeholders, and participation was not restricted. Over the study period, IAM responses (completed questionnaires) were collected using SurveyMonkey^®^ (10) in the secured INESSS database. The responses were anonymized and imported into a single Microsoft Excel spreadsheet (*Microsoft Corporation, 2018*) with columns named according to the questions and each row referring to a single response.

### Participants

The participants included all INESSS product users, consisting of four groups in accordance with previous INESSS reports (8).

- Group #1: Health and social services network members (e.g., health managers).
- Group #2: Health and social services professionals.
- Group #3: Patients, their relatives, caregivers, and the general public.
- Group #4: Students, teachers, and education-related participants (identified among the category “other”; specification provided).

The participants’ areas were asked about their region and categorized into 18 regions as defined by the Ministry of Health and Social Services (Supplementary Appendix 2).

There were three gender groups: women, men, and prefer not to answer. There were seven age groups, from 18 to 24, from 25 to 74 (divided into five groups of 10 years), and 75 years old and older. In order to reach a group of more than 200 respondents, we categorized 65-year-olds and older as one age group. For each specific analysis, some groups were disregarded.

### Intervention (products)

This study includes all products with or without recommendations assessed using the IAM questionnaire: In total, we gathered responses on various INESSS products, for example, clinical guidelines, states of practice, and states of knowledge.

### Ethical considerations

INESSS kept the gathered data confidential and protected it against unauthorized access. The participants’ responses were anonymized by INESSS before their transfer to the McGill research team. The protocol was revised by an external reviewer and was approved by the McGill institutional review board (IRB# A05-B52-22B).

### Statistical analysis

The conceptual framework supporting the analysis is presented in Supplementary Appendix 3 (11). The data set was provided in XLSX format, which was then converted to comma-separated values (CSV) and was loaded into RStudio. To perform a descriptive analysis of the demographic data, we used RStudio (12) with relevant packages to create figures and tables. For each group of participants, descriptive statistical analyses were carried out to compare the frequencies of the types of use, the types of products, and the participants’ characteristics in terms of gender, age, occupation, and region. For each question, absolute response rates were calculated, and relative response rates were reported (the total number of responses being the denominator). For instance, we calculated the “use” percentage in the following manner:

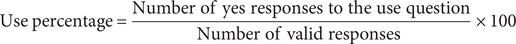

We compared the participants’ use of INESSS products among different groups of participants, their characteristics and the characteristics of the products, using Pearson’s Chi-Squared test (or Fisher’s exact test when applicable). We used RStudio (12) to test the statistical significance of the results. 99.5 percent confidence intervals were calculated for ordinal variables. Our experiences have shown that the classical *p*-value threshold of .05 is not strong enough to reliably draw conclusions in this context; therefore, as mentioned by Piroli, a *p*-value of .005 to be the cut-off point to reject the null hypothesis (13). Moreover, as explained in the missing values section, we calculated the *p*-value multiple times for some cases; therefore having a lower cut-off made our analysis more robust. To calculate the general relevance rates, satisfaction rates, and use rates of the products, we used the total number of valid responses as the denominator.

#### Invalid responses

**Figure 1.**
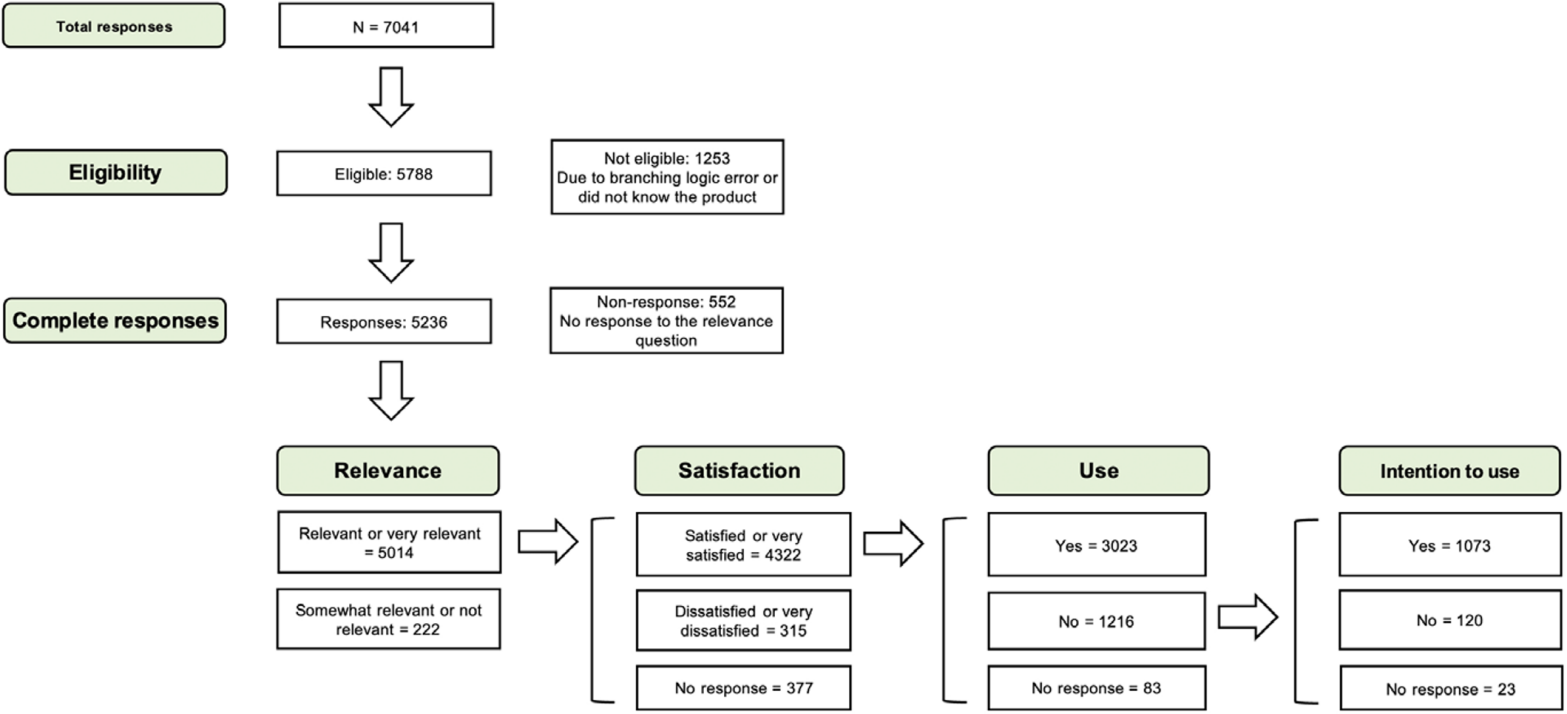
The number of responses to each question and the flow of responses and nonresponses. *Note*: The “no answer” counts are the total number of missing responses minus the responses from previous questions that did not transfer to the current question.

There were some groups of responses that were not eligible to be involved in the analysis and were therefore eliminated: (a) those who reported that they did not know the product when asked about the relevance, (b) due to a technical difficulty, some respondents had the option to answer the questionnaire before seeing the products. They mentioned this issue in the comments section. We found them during the qualitative analysis, and manually selected the participants’ ID numbers of those responses and removed them from the analysis in RStudio, (c) and responses that did not follow the branching logic of the questionnaire (considered as technical errors; see Figure 1 for the sequence of questions). To calculate the total percentages of relevance, satisfaction, use, and intention to use, we excluded the nonresponses to these questions. For instance, when measuring individual products’ use, we did not include responses that were missing the answer to the use question. When comparing the products, we did not include products with fewer than 30 responses for analysis.

#### Modification of variables

To simplify the calculations (binary), for relevance, we grouped “not relevant” and “somewhat relevant” as “No” and “relevant” and “very relevant” as “Yes.” Also for the satisfaction, we grouped “dissatisfied” and “very dissatisfied” as “No” and “satisfied” and “very satisfied” as “Yes.” We also found some responses recorded with more than one spelling variation and unified them.

#### Reclassification

Regarding inferential analysis, we grouped the responses based on professions, regions, and product properties (content). For instance, we divided the sample into two groups, one comprising healthcare professionals and another including all other professions (labeled as “other”), and compared usage across these two categories. To compare the use based on the regions’ population, we created two groups for regions based on their population. According to *Ministère de la Santé et des Services sociaux* (14) more than 60 percent of the population in Quebec lives in five regions (Montréal, Laval, Lanaudière, Laurentides, and Montérégie). So, we put the mentioned regions in one group and the other regions in another group and compared the use among them. We hypothesized that use might be higher in populated regions. We did the same analysis for regions without versus with medical universities (Montréal, Capitale-Nationale, Montérégie, and Estrie). For the analysis based on the content of products, we grouped them depending on whether they contained recommendations. Some products, such as states of practice and states of knowledge, do not have recommendations, but guidance, clinical guidelines, and quality standards always contain recommendations.

#### Missing values

We examined the data set for non-response errors and interruptions. In the case of missing data in an IAM response, incomplete cases were regarded as missing at random and were excluded pairwise. We planned to ask four questions sequentially. Basically, if the product was relevant to their context, they would be asked if they were satisfied; if they were satisfied, they would be asked about usage; and if they had not used the product before, they would be asked about their intention to use. For the missing data on satisfaction, use, and intention to use, we considered extreme scenarios where all the missing responses were positive or negative and compared the results of our analysis in two scenarios. We noticed that in some cases the *p*-value might have a range that included the significance cut-off, and consequently, we reported a range (an interval) for *p*-values that were close to .005.

## Results

We received a total of 7041 responses from 1 January 2021, to 31 December 2022. We removed noneligible and incomplete responses (see Figure 1). Therefore, we started the analysis with 5788 responses.

The flow of responses to the questions related to relevance, satisfaction, and use is shown in Figure 1. Regarding missing data, the numbers add up as we move through the branching logic. For satisfaction, we had 377 (377/5014 = 7.5%) missing responses, for use we had 460 (460/4699 = 9.8%), and for intention to use, we had 483 (483/1676 = 28.9%).

We examined the data for patterns in missing data that might be indicative of bias or technical issues in data gathering and did not find any. These patterns included missing answers to questions related to each other and missing data during the data collection period (Supplementary Appendix 4). As our main outcome, we focused on the use question. We also examined the data set for interruptions and abnormal patterns in missing data (see Supplementary Appendices 4 and 5).

### Respondent characteristics

Among the 5236 complete responses, 74.4 percent came from women, 24.4 percent from men, and 1.2 percent (61 individuals) who preferred not to say. Thirty-eight percent of responses were from participants who were aged 34 or younger, 48 percent were between 35 and 54, and the remaining 14 percent were 55 or older. 80.6 percent of responses were from healthcare professionals, 6 percent from patients, users, caregivers, and citizens, 4.8 percent from network managers, and 8.6 percent chose “other” or did not answer. The number of responses to each question is shown in Table 1.

**Table 1.** Characteristics of products and respondents and their responses

**Product types**	***n* (%)**	**Regions**	***n* (%)**
PC – Avis	292 (5.5%)	Abitibi-Témiscamingue	94 (1.8%)
PC – État de pratiques	135 (2.6%)	Bas-St-Laurent	253 (4.8%)
PC – État des connaissances	352 (6.7%)	Capitale-Nationale	723 (13.8%)
PC – Guides et normes	463 (8.8%)	Chaudière-Appalaches	249 (4.8%)
TC – Coup d’œil	132 (2.5%)	Estrie	320 (6.1%)
TC – Dépliant	189 (3.6%)	Lanaudière	230 (4.4%)
TC – Feuille de suivi	71 (1.4%)	Laurentides	288 (5.5%)
TC – Fiche synthèse	195 (3.7%)	Laval	200 (3.8%)
TC – Formulaire	1 (0.02%)	Mauricie-et-du-Centre-du-Québec	325 (6.2%)
TC – GUO	2355 (45.0%)	Montérégie	678 (12.9%)
TC – Outil aide décision	430 (8.2%)	Montréal	1275 (24.4%)
TC – Outil aide-mémoire	278 (5.3%)	Outaouais	165 (3.15%)
TC – Outil diagnostic	13 (0.25%)	Régions éloignées	218 (4.2%)
TC – Outil dialogue	12 (0.2%)	Saguenay-Lac-Saint-Jean	218 (4.2%)
TC – Outil interactif	15 (0.3%)	**Relevance**	***n***
TC – Outil réflexif	27 (0.5%)	Very relevant	3414
TC – PMN OC OIA	99 (1.9%)	Relevant	1600
TC – Repères	26 (0.5%)	Somewhat relevant	125
TC – Tutoriel	15 (0.3%)	Not relevant	97
TC – Vidéo	79 (1.5%)	**Satisfaction**	***n***
TC – Webinaire	57 (1.1%)	Very satisfied	1577
**Gender**	***n* (%)**	Satisfied	2745
Woman	3896 (74.4%)	Dissatisfied	87
Man	1279 (24.4%)	Very dissatisfied	228
Prefer not to answer	61 (1.2%)	Missing	377
**Age**	***n* (%)**	Not relevant (NA)	222
≤34	1990 (38.0%)	**Use**	***n***
35–54	2515 (48.0%)	Yes	3023
≥55	731 (14.0%)	No	1216
**Profession**	***n* (%)**	Missing	460
Healthcare professionals	4221 (80.6%)	Not relevant or not satisfied (NA)	537
Patient/user/family caregiver/citizen	314 (6.00%)	**Intention to use**	***n***
Managers	249 (4.76%)	Yes	1073
Other	452 (8.63%)	No	120
		Missing	483
		Not relevant or not satisfied or not used (NA)	3560

*Note*: For more information about INESSS product types please visit: https://numerique.banq.qc.ca/patrimoine/details/52327/4769647.

### Relevance, satisfaction, and use

**Figure 2.**
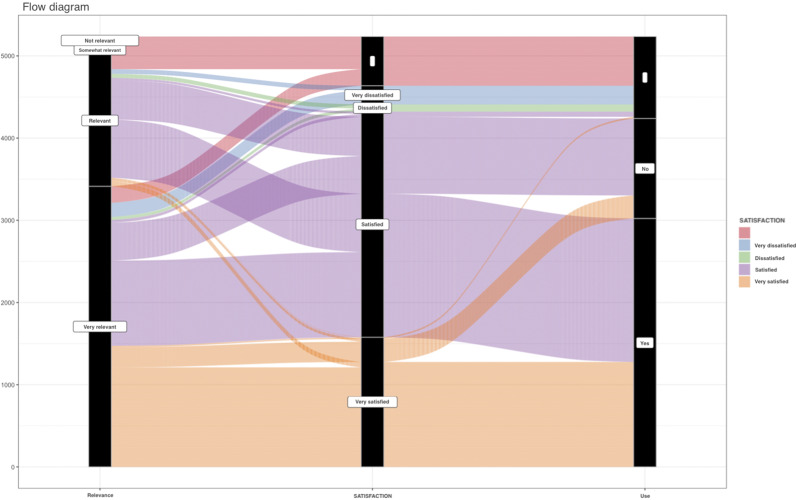
Flow of responses to questions regarding relevance, satisfaction, and use. Responses are grouped and shown in colored lanes based on the answer to the satisfaction question.

Of the 5236 complete responses, 95.8 percent (5014/5236) indicated that the product was relevant to their context; 82.5 percent (4322/5236) were satisfied with the product; 57.7 percent (3023/5236) reported that they had used the product; and 20.5 percent (1073/5236) reported that they had not yet used the product but intended to do so (Table 1). Taken together, 78.2 percent (4096/5236) either had used the INESSS product or intended to use it in the future. We also created a plot to visualize the flow of answers (see Figure 2). As shown, most of the participants were satisfied and used the products. Some of those who were satisfied used the products, and most of those who were very satisfied reported using the products.

#### Product use according to age

Results on the rate of product use are presented in Supplementary Appendix 6. Among the first age group (younger than 35-year-olds), 65.8 percent reported use (CI: 62.6–68.9); in the second age group (35- to 54-year-olds), 63.9 percent reported use (CI: 61.0–66.6); and in the last age group (55 and older), 54.0 percent (CI: 48.5–59.5). Therefore, we can see a pattern of decrease in use, and the *p*-value of the Fisher test for use among age groups was statistically significant (*p*-value <.0001). Supplementary Appendix 6 provides more information regarding relevance and satisfaction.

#### Product use according to profession

Among those respondents who answered the question about use, 65.6 percent (CI: 63.5–67.8) of healthcare professionals reported using the products, whereas 53.3 percent (CI: 48.6–57.9) of others reported use. The difference was statistically significant (*p*-value < .0001). For more details, see Supplementary Appendix 7.

#### Product use with or without recommendations

We compared the types of products based on whether they included recommendations or not. Products with recommendations were reported to be more useful than products without recommendations (see Supplementary Appendix 8). For products with recommendations, use was 65.2 percent (CI: 63.1–67.2), and for products without recommendations, use was 50.7 percent (CI: 45.1–56.3), and the chi-squared test was significant (*p*-value < .0001). A sub-analysis by profession (Supplementary Appendix 11) shows that this difference is driven mainly by healthcare professionals: 68 percent of professionals (*n* = 3471) reported using products with recommendations, compared with 49 percent (*n* = 395) for products without recommendations. Among other respondents (patients, caregivers, network managers, citizens), usage rates were identical at 53 percent for products both with (*n* = 684) and without recommendations (*n* = 226).

#### Product use according to regions

When comparing use between regions with medical universities (Montréal, Capitale-Nationale, Montérégie, and Estrie; see Supplementary Appendix 9), and other regions, we did not find any significant difference in reported use. Use was 64.1 percent (CI: 61.5–66.7) in regions with medical schools, and 62.2 percent (CI: 59.2–65.2) in other regions combined (*p*-value = .173; *p*-value range: .123–.358).

Among the five high-population regions (Montréal, Laval, Lanaudière, Laurentides, and Montérégie), the use percentage was 64.6 percent (CI: 61.9–67.3), compared with 61.9 percent (CI: 59.1–64.7) in the lower-population regions (see Supplementary Appendix 10). This difference was not statistically significant (*p* = .053; *p*-value range: .045–.106).

#### Use of recommendations across clinical topic-specific projects

Because products containing clinical recommendations were used more frequently, and the majority of responses centered on them, we only had sufficient data to evaluate topic-specific projects with recommendations (see Supplementary Appendix 8). Considering only projects with more than thirty responses, we calculated their use percentages and confidence intervals, and sorted the results by use in Supplementary Appendix 12. Of those, the three documents in the withdrawal and relapse product group received the greatest number of responses. Bronchitis, urinary infection, developmental language disorder, and cellulitis were among the most frequently used product groups, with usage rates ranging from 78.7 percent to 89.1 percent.

## Discussion

This prospective survey evaluated and demonstrated the use of INESSS knowledge products and the variability of INESSS stakeholders’ characteristics depending on the products during two consecutive years. We found that the majority (78.2 percent) of the respondents had used INESSS products before or intended to use them in the future. Based on our experience, all the general rates and percentages (relevance, satisfaction, and use) were above 50 percent and satisfactory (15;16). Among the respondents, 74.4 percent identified as women, and women were dominant in all professions, it was more prominent presence in the category of healthcare professionals. We are uncertain whether this imbalance stems from women being more likely to participate in surveys (17), or more inclined to seek health information (18;19). Taking the gender imbalance into consideration, the sample might not be representative of the general population; nonetheless, the sample size might still be representative of the study population (INESSS users). It was found that younger respondents were more likely to use the products. In other studies, younger physicians were more likely to adhere to guidelines and their practices were more affected by guidelines (20). The number of responses was higher for products with recommendations, and the likelihood of using these products was greater. Possibly, the higher usage of products with recommendations is due to the usage of healthcare professionals (as mentioned in the product use with or without recommendations section). We found that region did not have a significant effect on product use in general, but each product’s use in different regions should be studied separately. In this study, we focused on “use” and “intension to use” as the primary outcome (Supplementary Appendix 3). Although our prior mixed-methods review of guidelines from HTA organizations identified five sequential outcomes—(1) relevance, (2) cognitive or effective impact, (3) use in practice or for patients, and (4) and (5) individual/organizational health outcomes (21)—it would be difficult for survey respondents to accurately determine actual health benefits or broader system-level effects immediately after reading a guideline; therefore, because these outcomes are reached in order, we prioritized “use” as the most informative outcome in our survey, which presupposes relevance and cognitive/effective impact. Hence, we concentrated on measuring guideline use and intention to use, which were more directly assessable within the scope of this study.

### Limitations

We used an online web version of the questionnaire, which was the most suitable for our goals in this study; however, we acknowledge that a proportion of stakeholders might have mainly accessed INESSS products through their mobile application, or perhaps they might have accessed the offline version of the products downloaded before, and so did not have the opportunity to participate in the survey. Due to logistical constraints in the mobile deployment pipeline, we do not anticipate adding an in-app mobile survey for this project; future efforts by groups with appropriate resources may consider mobile-optimized surveys to better capture users who primarily interact via mobile. In some rare instances, there might have been multiple participations, that is, one participant assessing one product more than one time or assessing more than one product. The missing values were a minor limitation. We tried not to eliminate responses for this reason and considered two scenarios where all missing responses were from respondents who had positive or negative experiences. The missing values did not affect the reported significance of any analysis. Another limitation of this study is our decision to collapse both the Likert-scale items and the regional categories for analytical simplicity. First, converting four-category Likert scale responses into dichotomous outcomes can reduce the richness of the data. Although this approach facilitated clearer, more robust inferential statistics—particularly considering smaller cell counts in certain categories—it may have obscured nuanced differences in the degree of respondents’ relevance or satisfaction levels. Researchers employing larger data sets or different sampling strategies may benefit from preserving more response categories to capture subtler variations. Furthermore, our grouping of eighteen sociosanitary regions into binary categories was primarily driven by population distribution and the presence or absence of specific academic or institutional centers. We hypothesized that these factors might influence the level of use due to resource concentration and awareness. However, by doing so, we inevitably overlooked potential differences among urban, semi-urban, and rural areas, which can vary substantially in their healthcare infrastructures, professional networks, and access to information. Future investigations could address this gap by employing finer geographic or demographic stratifications (e.g., distinguishing semi-urban from predominantly rural areas), provided the sample sizes support such analyses.

## Conclusion

Most respondents, especially younger professionals, reported using or intending to use INESSS knowledge products, particularly products with recommendations. In our two-group regional analysis, usage rates did not differ significantly; however, this finding is preliminary, and finer geographic stratifications will be necessary before firm conclusions can be drawn about regional applicability. Within these methodological bounds, the present results help identify audiences for targeted dissemination, guide user-engagement strategies, and inform product refinement. Future work will pair the survey with qualitative methods and with more granular outcomes (such as cognitive or affective impact, practice-level changes, and patient-level or system-level effects) to deepen our understanding of how INESSS guidance translates into real-world benefit. The evaluative framework presented here can nevertheless serve as a practical template for other HTA agencies aiming to monitor and strengthen evidence uptake.

## Supporting information

10.1017/S0266462325103371.sm001 Baradaran et al. supplementary material Baradaran et al. supplementary material

## Data Availability

The R code supporting this study’s findings is available from the corresponding author upon reasonable request.

## Supplementary material

The supplementary material for this article can be found at http://doi.org/10.1017/S0266462325103371.
