# Supplementary material for: Use of knowledge translation products from health technology assessment: a prospective observational study
Source: Int J Technol Assess Health Care. 2026 Jan 9;42(1):e3. doi: 10.1017/S0266462325103371 (PMC12826861; doi:10.1017/S0266462325103371)
Supplement: Baradaran et al. supplementary material [file S0266462325103371sup001.zip › Appendix 2.docx]

| **APPENDIX 2. TABLE OF VARIABLES** | | | | | | | | | |
| --- | --- | --- | --- | --- | --- | --- | --- | --- | --- |
| **Variable** | **Definition** | **Values** | | | | | | | |
| Name | The topic of the products. | Titles of the products | | | | | | | |
| Type | The type of the document. | PC – Avis; PC – Guides et normes;PC – État des connnaisances; PC – État de pratiques; TC – GUO; TC – Fiche synthèse; TC – outil aide décision; TC – outil dialogue; TC – dépliant; TC – vidéo; TC – outil diagnostic; TC – feuille de suivi; TC – coup d’œil; TC – Repères; TC – outil interactif; TC – outil aide-mémoire | | | | | | | |
| Gender | How the users define their gender. | Woman, man, other, prefer not to say | | | | | | | |
| Age Group | Their age group | 18–24 | 25–34 | | 35–44 | 45–54 | 55–64 | | >65 |
| Profession | Their profession | Healthcare Professionals | | Patients, users, caregivers, citizens | | Network Managers | | Others | |
| Region | The Québec health regions were the user lives or works. | Bas-St-Laurent; Saguenay-Lac-Saint-Jean; Capitale-Nationale; Mauricie-et-du-Centre-du-Québec; Estrie; Montréal; Outaouais; Chaudière-Appalaches; Laval; Lanaudière; Laurentides; Montérégie; Nunavik; Terre-Cries-de-la-Baie-James; Abitibi-Témiscamingue; Côte-Nord; Nord-du-Québec; Gaspésie-îles-de-la-Madeleine | | | | | | | |
| Use | The document was used in past. | Yes | | | | No | | | |
| Intention to Use | The document was never used, but they are planning to use it in the future. | Yes | | | | No | | | |
| Recommendation | An indication that something is suitable for a particular purpose. | With recommendation | | | | Without recommendation | | | |
