# Supplementary material for: Use of knowledge translation products from health technology assessment: a prospective observational study
Source: Int J Technol Assess Health Care. 2026 Jan 9;42(1):e3. doi: 10.1017/S0266462325103371 (PMC12826861; doi:10.1017/S0266462325103371)
Supplement: Baradaran et al. supplementary material [file S0266462325103371sup001.zip › Appendix 3.docx]

**APPENDIX 3. CONCEPTUAL FRAMEWORK**

**
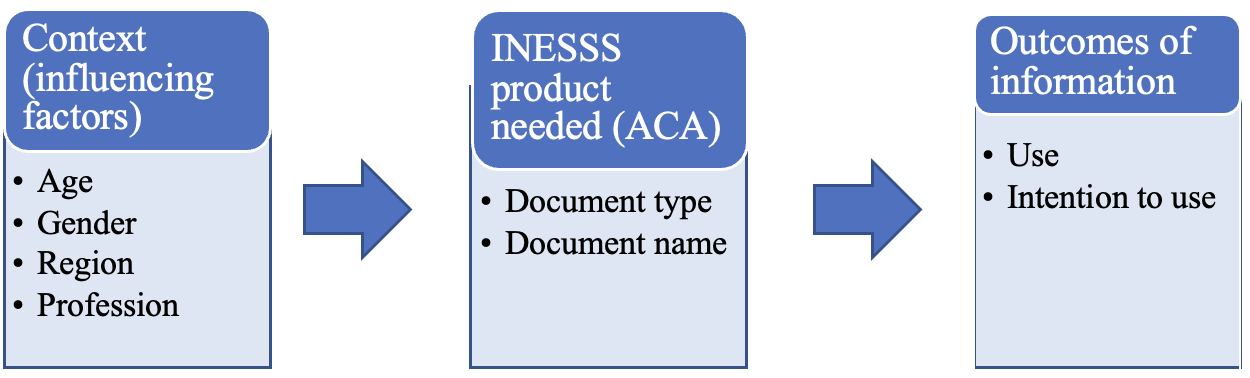
**

**Adapted from** Pluye, P., El Sherif, R., Granikov, V., Hong, Q.N., Vedel, I., Galvao, M.C.B., Frati, F.E., Desroches, S., Repchinsky, C., Rihoux, B., Légaré, F., Burnand, B., Bujold, M. and Grad, R. (2019), Health outcomes of online consumer health information: A systematic mixed studies review with framework synthesis. Journal of the Association for Information Science and Technology, 70: 643-659. <https://doi.org/10.1002/asi.24178>
