# Supplementary material for: Use of knowledge translation products from health technology assessment: a prospective observational study
Source: Int J Technol Assess Health Care. 2026 Jan 9;42(1):e3. doi: 10.1017/S0266462325103371 (PMC12826861; doi:10.1017/S0266462325103371)
Supplement: Baradaran et al. supplementary material [file S0266462325103371sup001.zip › Appendix 4.docx]

| **Appendix 4.** Variability of use during the data gathering period. | | | | | | | | | |
| --- | --- | --- | --- | --- | --- | --- | --- | --- | --- |
| **Time**  **Use** | **2021 Q1 (N=794)** | **2021 Q2 (N=756)** | **2021 Q3 (N=790)** | **2021 Q4 (N=634)** | **2022 Q1 (N=502)** | **2022 Q2 (N=628)** | **2022 Q3 (N=467)** | **2022 Q4 (N=665)** | **Overall (N=5236)** |
| **Missing** | 66 (8.31%) | 66 (8.73%) | 67 (8.48%) | 43 (6.78%) | 40 (7.97%) | 65 (10.4%) | 47 (10.1%) | 66 (9.92%) | 460 (8.79%) |
| **No** | 295 (37.2%) | 260 (34.4%) | 271 (34.3%) | 218 (34.4%) | 156 (31.1%) | 205 (32.6%) | 139 (29.8%) | 209 (31.4%) | 1753 (33.5%) |
| **Yes** | 433 (54.5%) | 430 (56.9%) | 452 (57.2%) | 373 (58.8%) | 306 (61.0%) | 358 (57.0%) | 281 (60.2%) | 390 (58.6%) | 3023 (57.7%) |

*Note: Collection was continuous across 2021 Q1–2022 Q4 (no quarters with zero records; N=467–794). “Use = Yes” varied 54.5%–61.0% (range 6.5 points), indicating no discontinuities or major variability.*
