# Supplementary material for: Use of knowledge translation products from health technology assessment: a prospective observational study
Source: Int J Technol Assess Health Care. 2026 Jan 9;42(1):e3. doi: 10.1017/S0266462325103371 (PMC12826861; doi:10.1017/S0266462325103371)
Supplement: Baradaran et al. supplementary material [file S0266462325103371sup001.zip › Appendix 5.docx]

*
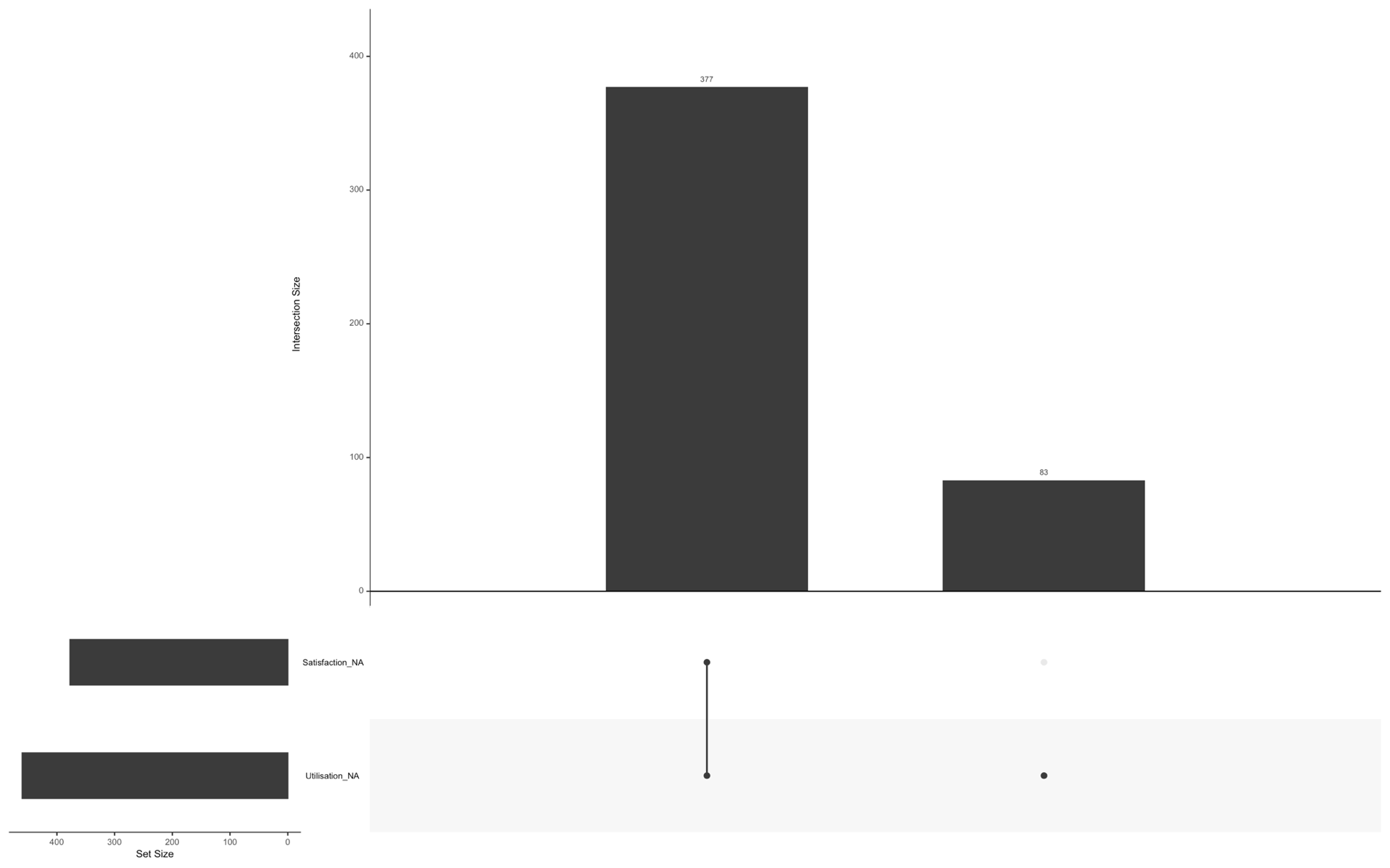
*

**Appendix 5.** Coincidence of missing responses. As expected, in some instances the answers to satisfaction, use, and intention to use questions were missing concurrently. There was no irregularity in the missing data.
