# Supplementary material for: Use of knowledge translation products from health technology assessment: a prospective observational study
Source: Int J Technol Assess Health Care. 2026 Jan 9;42(1):e3. doi: 10.1017/S0266462325103371 (PMC12826861; doi:10.1017/S0266462325103371)
Supplement: Baradaran et al. supplementary material [file S0266462325103371sup001.zip › Appendix 6.docx]

| **Appendix 6.** Use among different age groups. | | | | |
| --- | --- | --- | --- | --- |
|  | **≤34** | **35–54** | **≥55** | **Overall** |
|  | **(N=1834)** | **(N=2300)** | **(N=642)** | **(N=4776)** |
| **Relevance** | | | | |
| No | 74 (4.0%) | 93 (4.04%) | 55 (8.6%) | 222 (4.65%) |
| Yes | 1760 (96.0%) | 2207 (96.0%) | 587 (91.4%) | 4554 (95.4%) |
| **Satisfaction** | |  |  |  |
| No | 182 (9.9%) | 238 (10.3%) | 117 (18.2%) | 537 (11.2%) |
| Yes | 1652 (90.1%) | 2062 (89.7%) | 525 (81.8%) | 4239 (88.8%) |
| **Use** | |  |  |  |
| No | 627 (34.2%) | 831 (36.1%) | 295 (46.0%) | 1753 (36.7%) |
| Yes | 1207 (65.8%) | 1469 (63.9%) | 347 (54.0%) | 3023 (63.3%) |
