# Supplementary material for: Use of knowledge translation products from health technology assessment: a prospective observational study
Source: Int J Technol Assess Health Care. 2026 Jan 9;42(1):e3. doi: 10.1017/S0266462325103371 (PMC12826861; doi:10.1017/S0266462325103371)
Supplement: Baradaran et al. supplementary material [file S0266462325103371sup001.zip › Appendix 7.docx]

| **Appendix 7.** Use among health care professionals and others. | | | |
| --- | --- | --- | --- |
|  | **Healthcare professionals** | **Others** | **Overall** |
|  | **(N=3866)** | **(N=910)** | **(N=4776)** |
| **Relevance** | | | |
| No | 108 (2.8%) | 114 (12.5%) | 222 (4.6%) |
| Yes | 3758 (97.2%) | 796 (87.5%) | 4554 (95.4%) |
| **Satisfaction** | |  |  |
| No | 361 (9.3%) | 176 (19.3%) | 537 (11.2%) |
| Yes | 3505 (90.7%) | 734 (80.7%) | 4239 (88.8%) |
| **Use** | |  |  |
| No | 1328 (34.4%) | 425 (46.7%) | 1753 (36.7%) |
| Yes | 2538 (65.6%) | 485 (53.3%) | 3023 (63.3%) |
