# Supplementary material for: Use of knowledge translation products from health technology assessment: a prospective observational study
Source: Int J Technol Assess Health Care. 2026 Jan 9;42(1):e3. doi: 10.1017/S0266462325103371 (PMC12826861; doi:10.1017/S0266462325103371)
Supplement: Baradaran et al. supplementary material [file S0266462325103371sup001.zip › Appendix 8.docx]

| **Appendix 8.** Use based on the content of products. | | | |
| --- | --- | --- | --- |
|  | **Products without recommendations** | **Products containing recommendations** | **Overall** |
|  | **(N=621)** | **(N=4155)** | **(N=4776)** |
| **Relevance** | | | |
| No | 41 (6.6%) | 181 (4.36%) | 222 (4.6%) |
| Yes | 580 (93.4%) | 3974 (95.6%) | 4554 (95.4%) |
| **Satisfaction** | |  |  |
| No | 83 (13.4%) | 454 (10.9%) | 537 (11.2%) |
| Yes | 538 (86.6%) | 3701 (89.1%) | 4239 (88.8%) |
| **Use** | |  |  |
| No | 306 (49.3%) | 1447 (34.8%) | 1753 (36.7%) |
| Yes | 315 (50.7%) | 2708 (65.2%) | 3023 (63.3%) |
