# Supplementary material for: Use of knowledge translation products from health technology assessment: a prospective observational study
Source: Int J Technol Assess Health Care. 2026 Jan 9;42(1):e3. doi: 10.1017/S0266462325103371 (PMC12826861; doi:10.1017/S0266462325103371)
Supplement: Baradaran et al. supplementary material [file S0266462325103371sup001.zip › Appendix 9.docx]

| **Appendix 9.** Use based on region (with vs. without faculties of medicine). | | | |
| --- | --- | --- | --- |
|  | **Regions with faculties of medicine** | **Other regions** | **Overall** |
|  | **(N=2723)** | **(N=2053)** | **(N=4776)** |
| **Relevance** | | | |
| No | 117 (4.30%) | 105 (5.11%) | 222 (4.65%) |
| Yes | 2606 (95.7%) | 1948 (94.9%) | 4554 (95.4%) |
| **Satisfaction** | |  |  |
| No | 294 (10.8%) | 243 (11.8%) | 537 (11.2%) |
| Yes | 2429 (89.2%) | 1810 (88.2%) | 4239 (88.8%) |
| **Use** | |  |  |
| No | 977 (35.9%) | 776 (37.8%) | 1753 (36.7%) |
| Yes | 1746 (64.1%) | 1277 (62.2%) | 3023 (63.3%) |
