# Supplementary material for: Use of knowledge translation products from health technology assessment: a prospective observational study
Source: Int J Technol Assess Health Care. 2026 Jan 9;42(1):e3. doi: 10.1017/S0266462325103371 (PMC12826861; doi:10.1017/S0266462325103371)
Supplement: Baradaran et al. supplementary material [file S0266462325103371sup001.zip › Appendix 10.docx]

| **Appendix 10.** Use based on region (populated regions vs. not populated). | | | |
| --- | --- | --- | --- |
|  | **Populated** | **Not populated** | **Overall** |
|  | **(N=2431)** | **(N=2345)** | **(N=4776)** |
| **Relevance** | | | |
| No | 90 (3.7%) | 132 (5.63%) | 222 (4.6%) |
| Yes | 2341 (96.3%) | 2213 (94.4%) | 4554 (95.4%) |
| **Satisfaction** | |  |  |
| No | 244 (10.0%) | 293 (12.5%) | 537 (11.2%) |
| Yes | 2187 (90.0%) | 2052 (87.5%) | 4239 (88.8%) |
| **Use** | |  |  |
| No | 860 (35.4%) | 893 (38.1%) | 1753 (36.7%) |
| Yes | 1571 (64.6%) | 1452 (61.9%) | 3023 (63.3%) |
