# Supplementary material for: Use of knowledge translation products from health technology assessment: a prospective observational study
Source: Int J Technol Assess Health Care. 2026 Jan 9;42(1):e3. doi: 10.1017/S0266462325103371 (PMC12826861; doi:10.1017/S0266462325103371)
Supplement: Baradaran et al. supplementary material [file S0266462325103371sup001.zip › Appendix 11.docx]

| **Appendix 11.** Relative usage rates based on profession and content of the products. | | |
| --- | --- | --- |
| **Products**  **Profession** | **Without recommendation** | **With recommendation** |
| **Healthcare professionals** | 0.49 (195/395) | 0.68 (2343/3471) |
| **Others** | 0.53 (120/226) | 0.53 (365/684) |

*Note: For comparison purposes, the use rate is calculated only based on the answer to the use question (yes/yes+no). The gap between usage of products with and without recommendations is more notable among healthcare professionals.*
