# Supplementary material for: Use of knowledge translation products from health technology assessment: a prospective observational study
Source: Int J Technol Assess Health Care. 2026 Jan 9;42(1):e3. doi: 10.1017/S0266462325103371 (PMC12826861; doi:10.1017/S0266462325103371)
Supplement: Baradaran et al. supplementary material [file S0266462325103371sup001.zip › Appendix 12.docx]

| **Appendix 12.** Use percentage of the most popular projects with recommendations. | | | | |
| --- | --- | --- | --- | --- |
| **Project name *** | **Types of products (n)** | **Total responses (n)** | **Use (%)** | **Use CI (%)** |
| Bronchite (Bronchitis) | 2 | 64 | 89.1 | 72.4 - 96.2 |
| Infection urinaire (Urinary tract infection) | 2 | 240 | 80.8 | 72.7 - 87 |
| Trouble développemental du langage (Developmental language disorder) | 3 | 34 | 79.4 | 53.5 - 92.8 |
| Cellulite (Cellulitis) | 3 | 268 | 78.7 | 70.9 - 84.9 |
| C. Difficile | 1 | 46 | 78.3 | 56.6 - 90.9 |
| Antidiabétique (Antidiabetic) | 1 | 52 | 75 | 54.7 - 88.2 |
| Anticoagulant | 2 | 40 | 75 | 51.5 - 89.4 |
| Pneumonie (Pneumonia) | 3 | 196 | 74.5 | 64.8 - 82.2 |
| TCCL (Mild traumatic brain injury / concussion) | 4 | 264 | 73.5 | 65.2 - 80.4 |
| Anticoagulant_warfarine (Anticoagulant-Warfarin) | 4 | 90 | 72.2 | 57.2 - 83.5 |
| Dépistage cancer de la prostate (Prostate cancer screening) | 2 | 35 | 71.4 | 46.3 - 87.9 |
| Chlamydia (Chlamydia) | 2 | 122 | 70.5 | 57.7 - 80.7 |
| Rhino (Common cold / Viral rhinitis / acute viral upper‑respiratory infection) | 2 | 46 | 69.6 | 47.9 - 85 |
| MPOC - Maladie pulmonaire obstructive chronique  (Chronic obstructive pulmonary disease) | 6 | 68 | 69.1 | 51.6 - 82.5 |
| Influenza | 2 | 63 | 68.3 | 50 - 82.2 |
| Statine (Statin) | 5 | 169 | 66.9 | 56 - 76.2 |
| TCCMG (Moderate‑to‑severe traumatic brain injury) | 5 | 68 | 66.2 | 48.6 - 80.2 |
| Bêta lactamine (Beta‑lactam antibiotics) | 3 | 76 | 65.8 | 49.3 - 79.2 |
| Condylome (Condyloma / genital warts) | 2 | 54 | 64.8 | 45.1 - 80.5 |
| Trouble usage opioids (Opioid use disorder) | 4 | 354 | 60.2 | 52.7 - 67.2 |
| Lyme (Lyme disease) | 11 | 183 | 59 | 48.5 - 68.7 |
| Contraception orale (Oral contraception) | 1 | 48 | 58.3 | 37.9 - 76.3 |
| Sevrage et Rechute (Withdrawal and relapse) | 3 | 478 | 57.7 | 51.3 - 63.9 |
| Déficience intellectuelle (Intellectual disability) | 2 | 50 | 54 | 34.4 - 72.4 |
| Echocardiographie (Echocardiography) | 3 | 48 | 52.1 | 32.4 - 71.2 |
| Mycoplasma (Mycoplasma infection) | 3 | 287 | 48.8 | 40.6 - 57 |
| RCR – Naloxone (CPR – Naloxone) | 2 | 31 | 41.9 | 20.3 - 67.1 |
| Rééducation périnéale et pelvienne (Pelvic and perineal rehabilitation) | 1 | 33 | 36.4 | 16.9 - 61.6 |

* All English translations of the original French titles (appearing in parentheses) are by the authors.
