# Supplementary figures and images for: Use of knowledge translation products from health technology assessment: a prospective observational study
Source: Int J Technol Assess Health Care. 2026 Jan 9;42(1):e3. doi: 10.1017/S0266462325103371 (PMC12826861; doi:10.1017/S0266462325103371)

**APPENDIX 1. IAM-INESSS-2019 QUESTIONNAIRE**

| 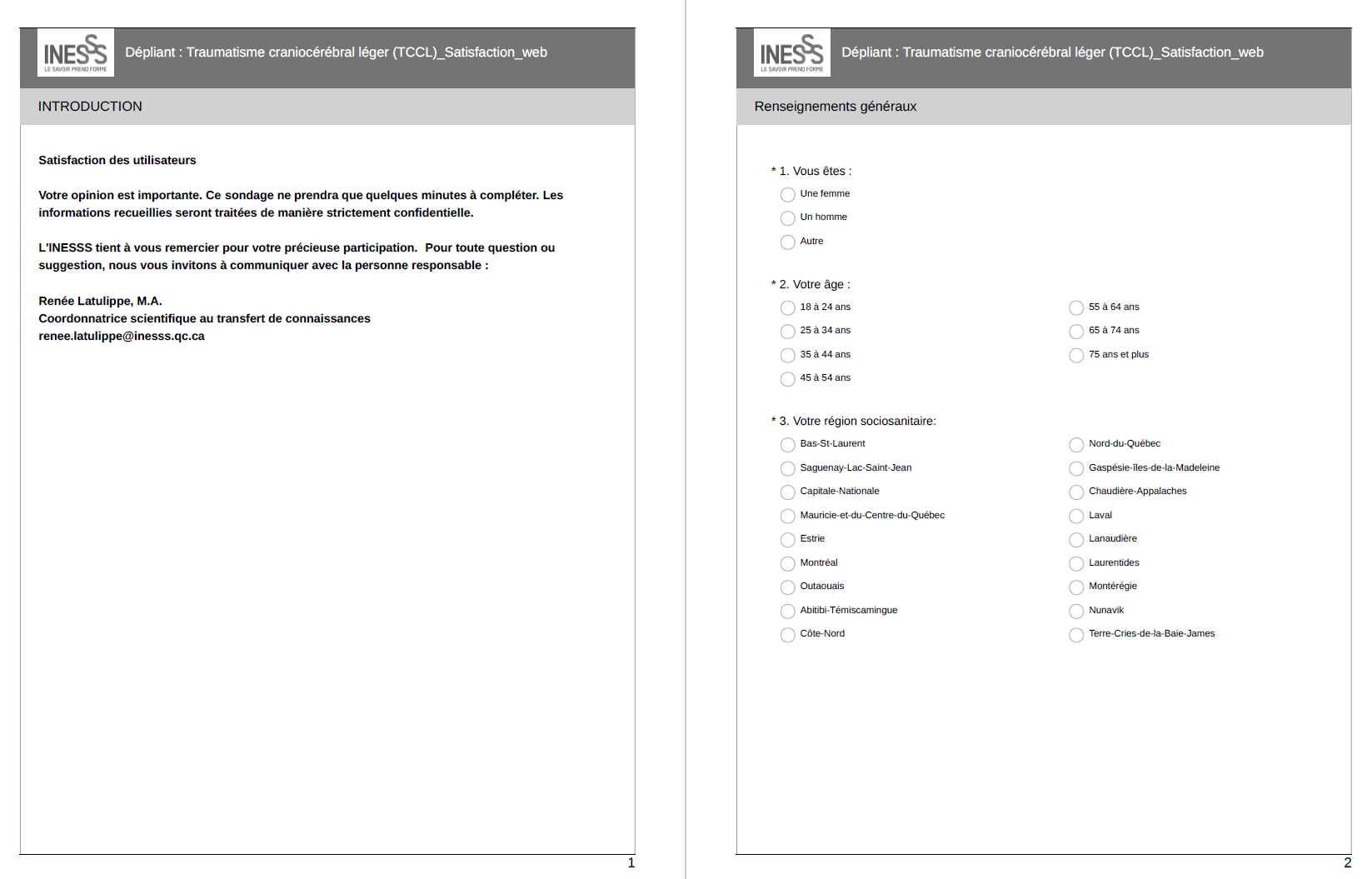 |
| --- |
| 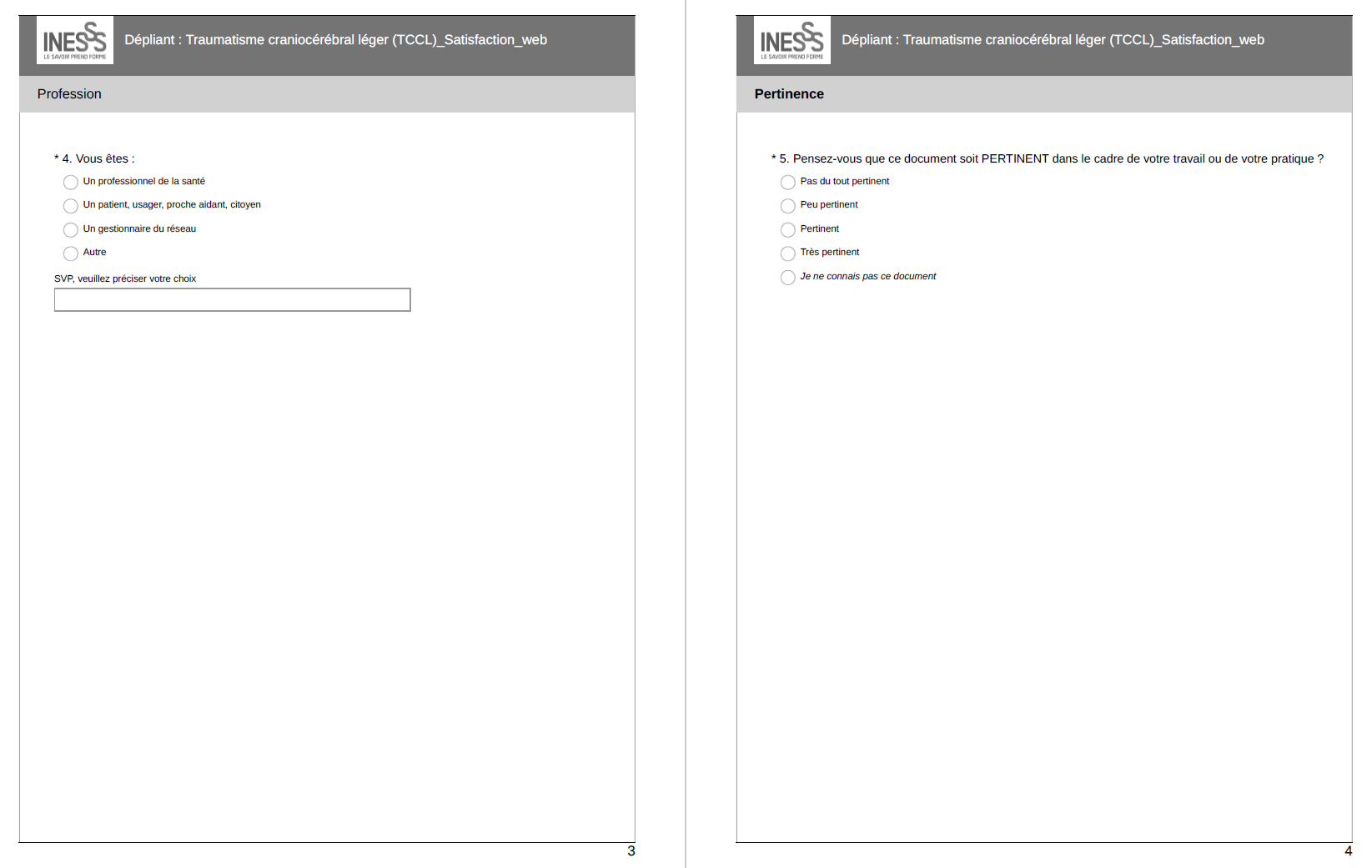 |
| 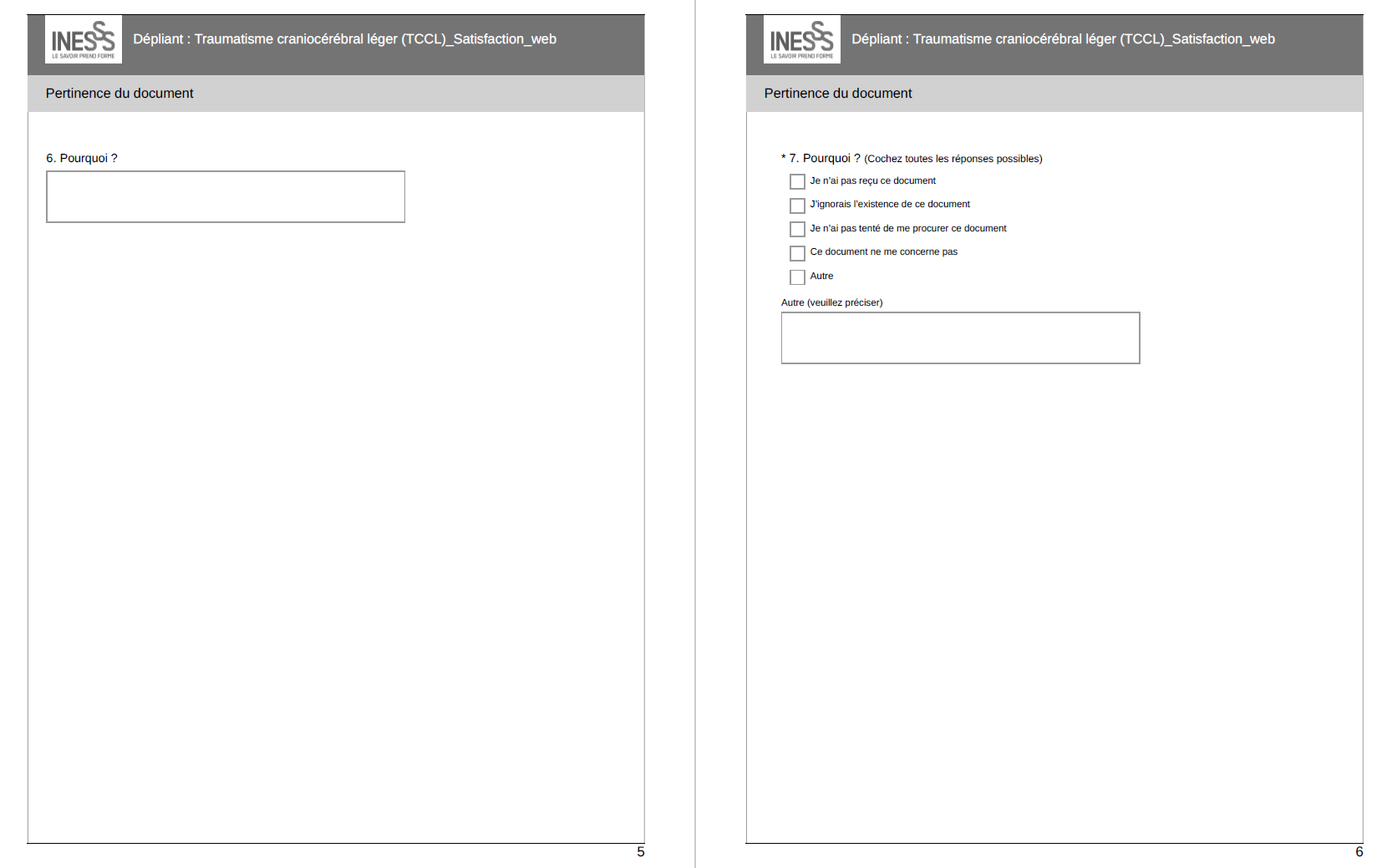 |
| 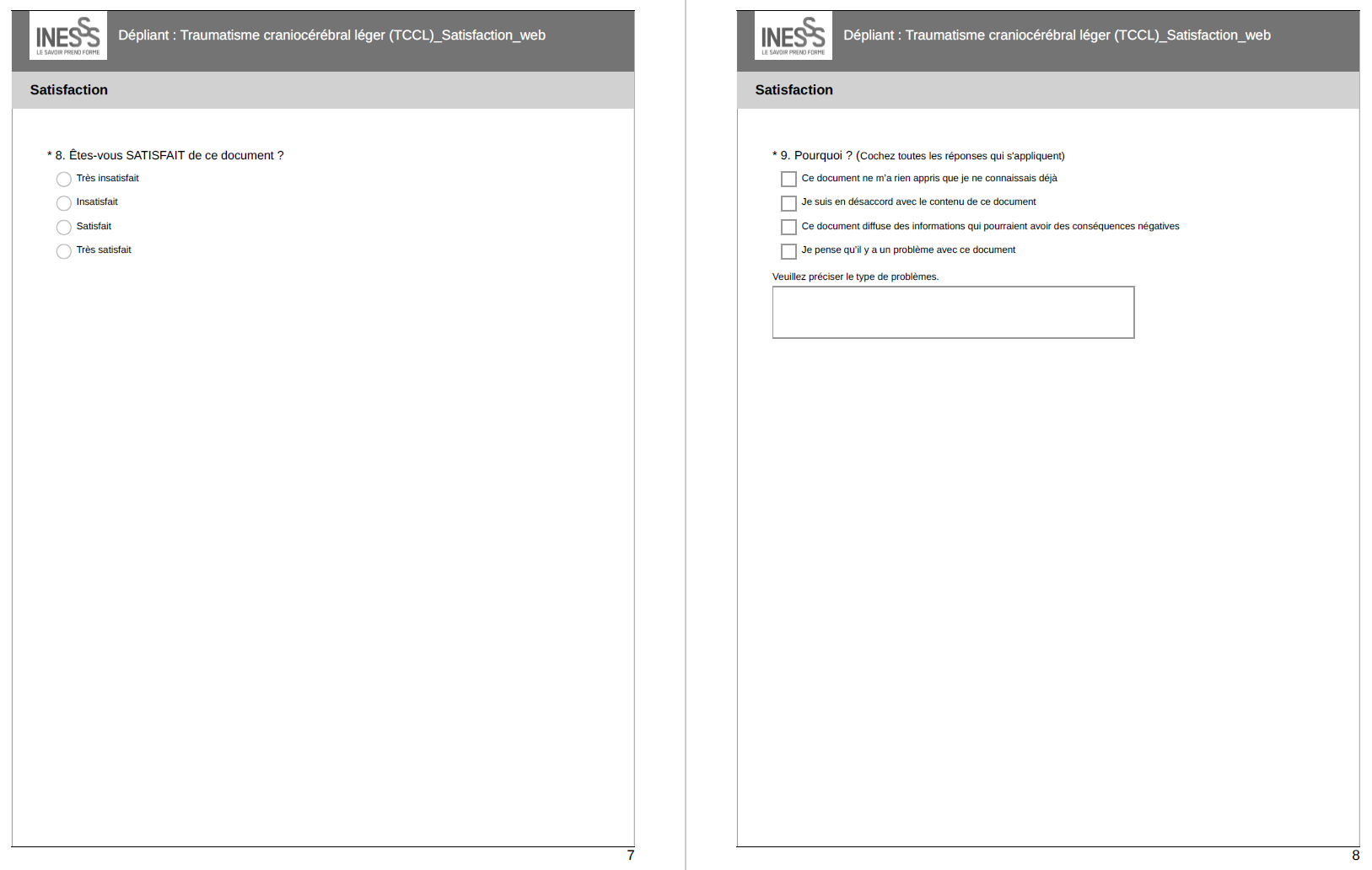 |
| 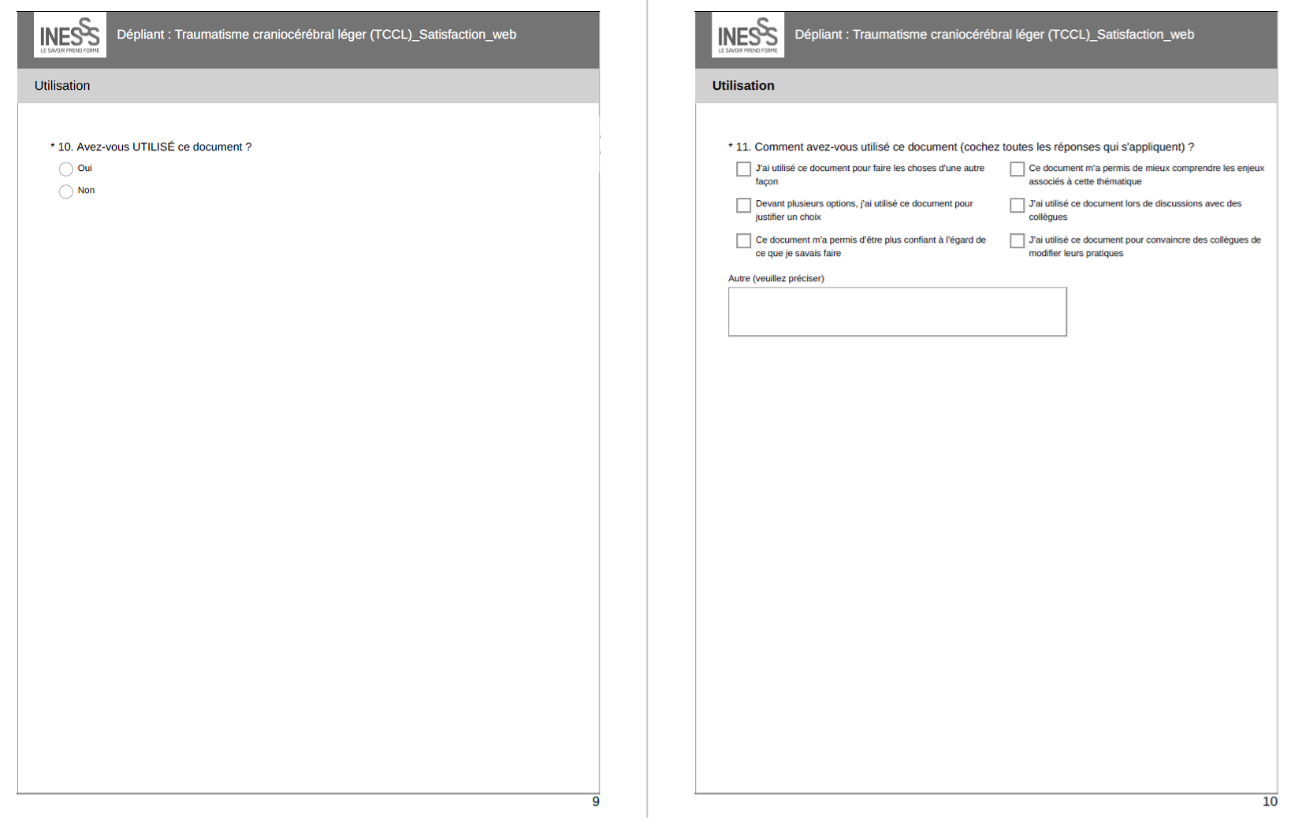 |
| 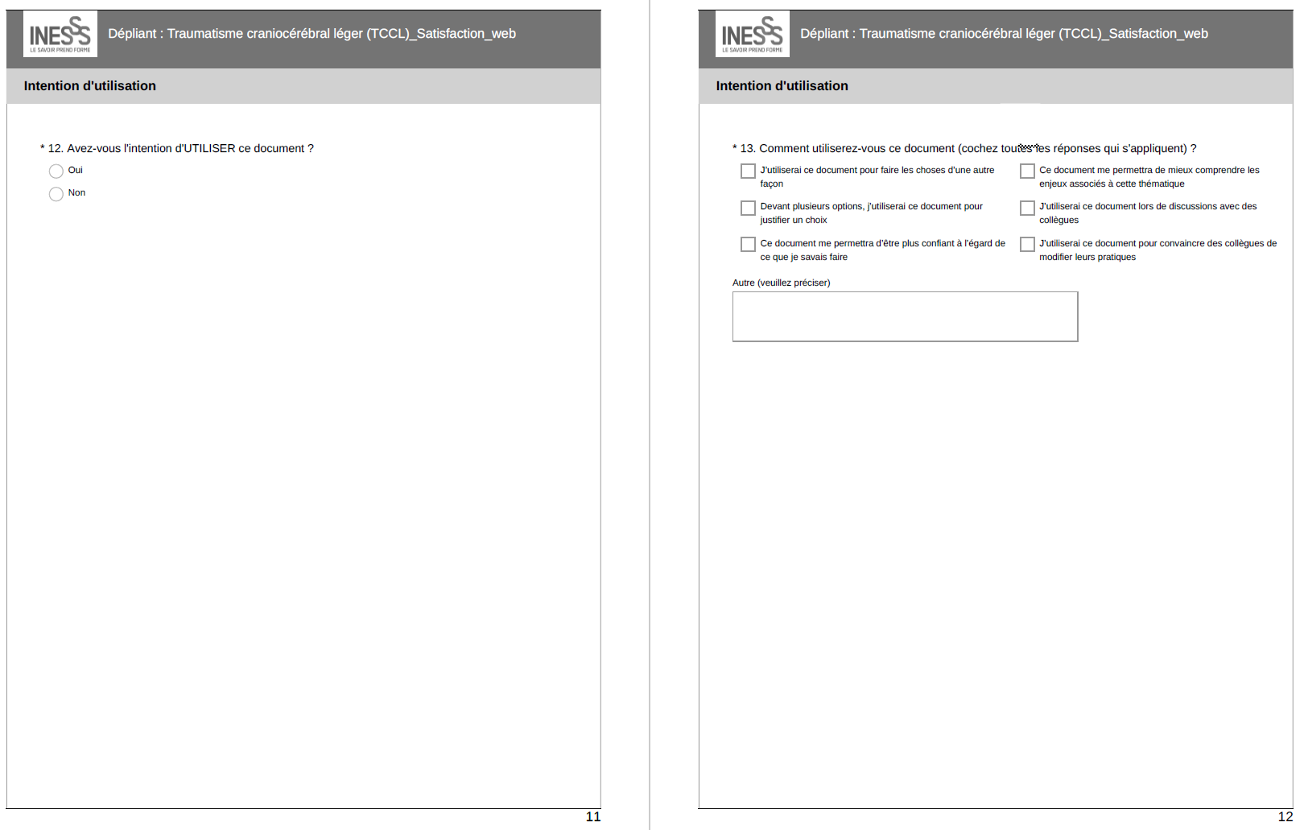 |
| 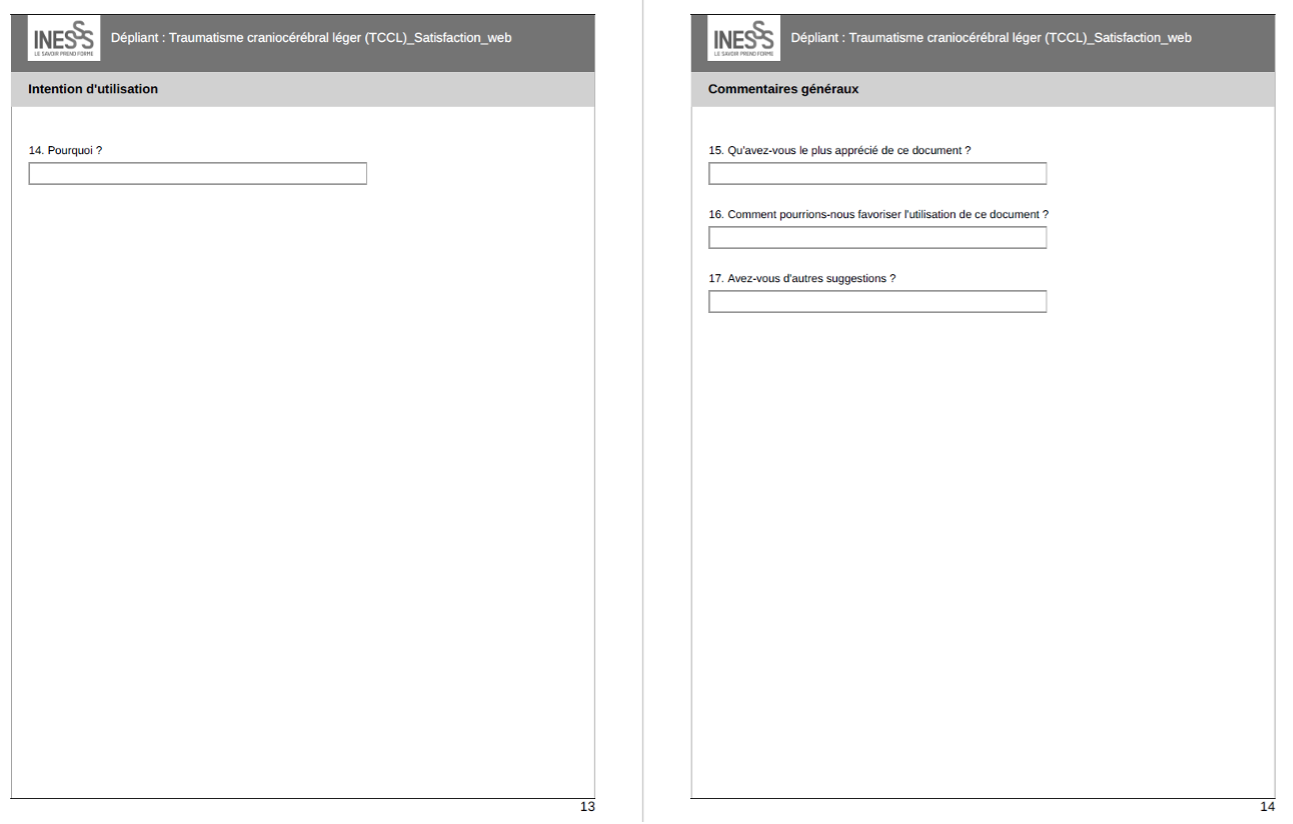 |
| 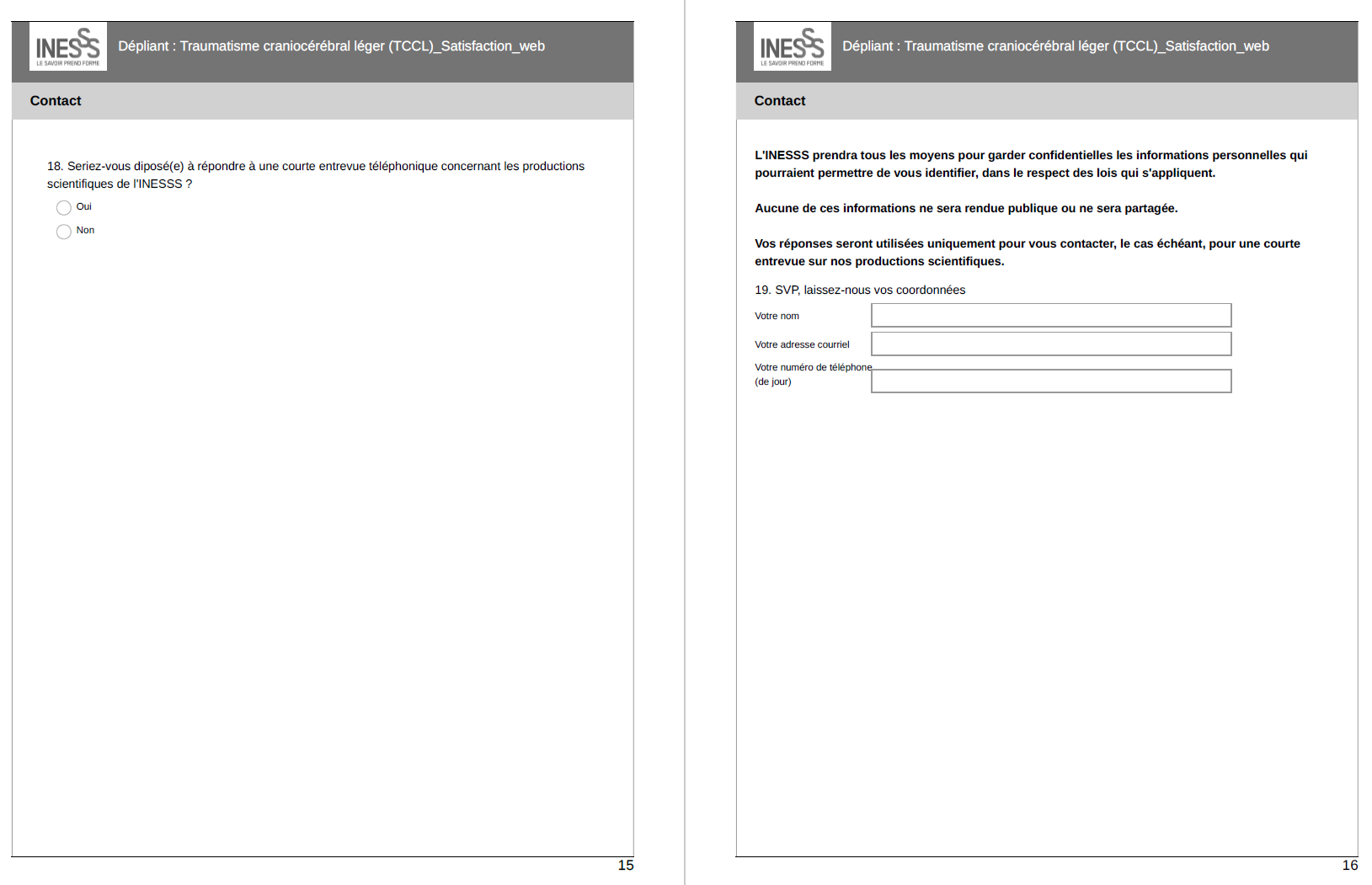 |
| 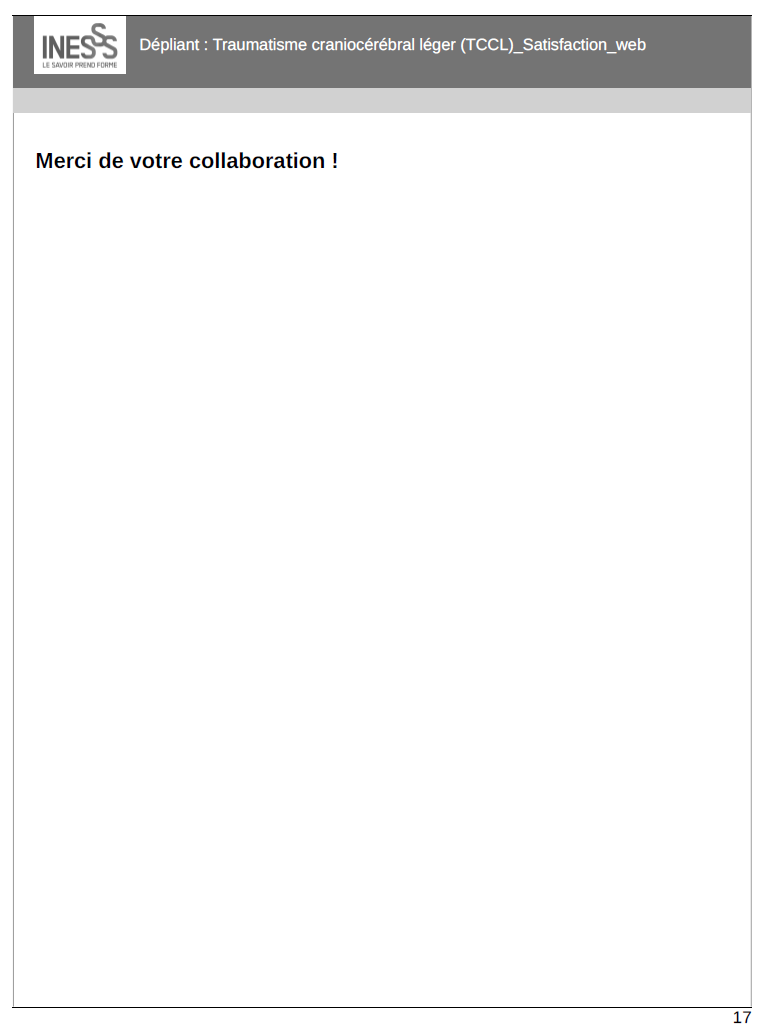 |

Supplement: Baradaran et al. supplementary material [file S0266462325103371sup001.zip › Appendix 1.docx]
